# Authors’ Reply to: Additional Considerations for US Residency Selection After Pass/Fail USMLE Step 1. Comment on “The US Residency Selection Process After the United States Medical Licensing Examination Step 1 Pass/Fail Change: Overview for Applicants and Educators”

**DOI:** 10.2196/50109

**Published:** 2023-08-17

**Authors:** Ahmad Ozair, Vivek Bhat, Donald K E Detchou

**Affiliations:** 1 Bloomberg School of Public Health Johns Hopkins University Baltimore, MD United States; 2 Miami Cancer Institute Baptist Health South Florida Miami, FL United States; 3 St. John’s Medical College Bangalore India; 4 Perelman School of Medicine University of Pennsylvania Philadelphia, PA United States; 5 Department of Neurosurgery Hospital of the University of Pennsylvania Philadelphia, PA United States

**Keywords:** admission, assessment, postgraduate training, selection, standardized testing, graduate medical education, medical education

We appreciate the thoughtful correspondence by Sow et al [1] in response to our work [2] and discuss further considerations below.

Sow et al [1] have highlighted the sociocultural and ethical challenges surrounding unpaid research fellowships, pursued not only by international medical graduates (IMGs) but increasingly by MD and DO students in the United States as well. We have discussed this issue before, highlighting that IMG aspiring for several competitive specialties pursue several postdoctoral research years, although quantitative data remain unavailable [3]. The USMLE (United States Medical Licensing Examination) Step 1 pass/fail change has occurred notwithstanding a substantial supply-demand mismatch in competitive specialties, which has historically warranted and continues to warrant measures (like USMLE scores) to facilitate the rank-ordering of applicants. Program rank lists require an ever-increasing number of applicants per position to be assessed and objectively ranked [4]. Therefore, research fellowships will likely be increasingly pursued to demonstrate academic accomplishment, given the loss of major objective metrics like the USMLE Step 1 score, which we have highlighted previously [3].

Several publications have indicated the presence of elements of socioeconomic disparity, racial and/or ethnic bias, or financial privilege in USMLE. We argue this is potentially true for nearly all other components of the residency evaluation process. It is contended that comprehensive USMLE preparation forces students to use expensive preparatory resources. What is frequently unstated here is the often exponentially greater cost of unpaid research years, unpaid volunteering, and away rotations, the latter typically unpaid.

Therefore, analyses have long been warranted to identify the components of the residency application that most perpetuate existing disparities. While the quantitative nature of USMLE scores permits easy analyses of correlation and association (using multivariable regression) with sociodemographic and ethnoracial variables, the subjective nature of the other components of residency evaluation prohibits the ease, accessibility, and rapidity of such analyses. To illustrate, it is manually challenging to thematically evaluate the tens of thousands of letters of recommendation submitted each year and assign them numeric scores to facilitate correlative analyses with sociodemographic variables. Such time-intensive processes have not been performed for each component of the residency evaluation process, including the medical school transcript, the “meaningful experiences” section, the personal statement, and the publication portfolio, among others.

Assigning numeric scoring to all subjective components of the residency application and then adding these hitherto unconsidered variables to multivariate regression analyses on USMLE scores would reduce confounding and determine which components likely represent the most amount of socioeconomic or ethnoracial bias. The rapidly evolving quality of large language models, including GPT-4 (OpenAI) and Bard (Google), permits automated qualitative analyses of subjective application materials of thousands of candidates, which will be critical for identifying the least biased application components. We predict this will likely redeem USMLE scores, given that a landmark blinded analysis of >5000 applications demonstrated that physical attractiveness outperformed class rank, clerkship grading, and Alpha Omega Alpha status for predicting interview desirability, but came second only to the USMLE Step 1 score [5].

Finally, several publications have stated unpaid research to be unjust [6]. Sow et al [1] in response to our work argued for increasing paid fellowships. An increase, while ideal, remains unlikely given the widespread financial pressures on academic medical systems. Persistence of the current unfavorable status quo will continue to necessitate unpaid research by IMGs as a stepping stone for competitive specialties.

## Abbreviations

IMG: international medical graduate
USMLE: United States Medical Licensing Examination
